# *In vitro* Effect of Harmine Alkaloid and Its *N*-Methyl Derivatives Against *Toxoplasma gondii*

**DOI:** 10.3389/fmicb.2021.716534

**Published:** 2021-08-05

**Authors:** Maria L. Alomar, Juan G. Yañuk, Sergio O. Angel, M. Micaela Gonzalez, Franco M. Cabrerizo

**Affiliations:** ^1^Laboratorio de Fotoquímica y Fotobiología Molecular, Instituto Tecnológico de Chascomús (INTECH), Universidad Nacional de San Martín (UNSAM) – Consejo Nacional de Investigaciones Científicas y Técnicas (CONICET), Chascomús, Argentina; ^2^Laboratorio de Parasitología Molecular, INTECH, UNSAM – CONICET, Chascomús, Argentina

**Keywords:** β-carbolines, 9-methyl-harmine, 2-methyl-harminium, toxoplasmosis, cell cycle arrest, γH2A.X

## Abstract

Toxoplasmosis is one of the most prevalent and neglected zoonotic global diseases caused by *Toxoplasma gondii*. The current pharmacological treatments show clinical limitations, and therefore, the search for new drugs is an urgent need in order to eradicate this infection. Due to their intrinsic biological activities, β-carboline (βC) alkaloids might represent a good alternative that deserves further investigations. In this context, the *in vitro* anti-*T. gondii* activity of three βCs, harmine (**1**), 2-methyl-harminium (**2**), and 9-methyl-harmine (**3**), was evaluated herein. Briefly, the three alkaloids exerted direct effects on the parasite invasion and/or replication capability. Replication rates of intracellular treated tachyzoites were also affected in a dose-dependent manner, at noncytotoxic concentrations for host cells. Additionally, cell cycle analysis revealed that both methyl-derivatives **2** and **3** induce parasite arrest in S/M phases. Compound **3** showed the highest irreversible parasite growth inhibition, with a half maximal inhibitory concentration (IC50) value of 1.8 ± 0.2 μM and a selectivity index (SI) of 17.2 at 4 days post infection. Due to high replication rates, tachyzoites are frequently subjected to DNA double-strand breaks (DSBs). This highly toxic lesion triggers a series of DNA damage response reactions, starting with a kinase cascade that phosphorylates a large number of substrates, including the histone H2A.X to lead the early DSB marker γH2A.X. Western blot studies showed that basal expression of γH2A.X was reduced in the presence of **3**. Interestingly, the typical increase in γH2A.X levels produced by camptothecin (CPT), a drug that generates DSB, was not observed when CPT was co-administered with **3**. These findings suggest that **3** might disrupt *Toxoplasma* DNA damage response.

## Introduction

*Toxoplasma gondii* is an obligate intracellular protozoan parasite that belongs to the phylum Apicomplexa, which infects a wide range of warm-blooded animals, causing toxoplasmosis. This is one of the most prevalent infections among humans (Flegr et al., 2014). Its apparent success or high prevalence is a direct consequence of the quite high infection rate of parasites, as well as its benign coexistence with immunocompetent hosts and the large distribution without geographical or climatic barriers (Carruthers, 1999).

Toxoplasmosis is usually asymptomatic in immunocompetent persons (over 80% of primary cases) (Alday and Doggett, 2017) and leads to chronic infection, with cyst formation mainly in the central nervous system (CNS) (Martins-Duarte et al., 2006). Symptoms may include bilateral cervical lymphadenopathy, accompanied by low-grade fever, and usually, treatment is not required (Durlach et al., 2003). However, it is noteworthy that ocular toxoplasmosis is an important cause of ocular impairment in immunocompetent persons, being one of the most frequent etiologies of posterior uveitis (de la Torre et al., 2014). In patients with impaired cellular immunity, i.e., patients with AIDS, with cancer, or under immunosuppressive treatments, toxoplasmosis requires lifelong therapy to control progressive infection and prevent relapse (Dubey and Jones, 2008; Martynowicz et al., 2020). In this respect, the primo-infection or the reactivation of a chronic *T. gondii* infection can cause neurological, systemic, and ocular diseases, with multifocal necrotizing encephalitis being the predominant manifestation (Rajapakse et al., 2013). In congenitally acquired toxoplasmosis, the severity of the symptoms depends on the gestational age at the time of maternal infection. Clinical manifestations might include chorioretinitis, blindness, mental or psychomotor retardation, intracranial calcifications, encephalitis, hydrocephalus, and even death (Belk et al., 2018).

The gold standard for treating acute toxoplasmosis is a combination of sulfadiazine (SDZ) and pyrimethamine (Araujo-Silva et al., 2020). However, these two drugs may produce severe side effects including thrombocytopenia, leucopenia, neutropenia (Rajapakse et al., 2013; Montazeri et al., 2018), and other adverse reactions such as agranulocytosis, toxic epidermal necrolysis (McLeod et al., 2006), allergy, and hepatic and renal complications (Mui et al., 2005). In addition, clarithromycin, azithromycin, spiramycin, and atovaquone have also been used for clinical toxoplasmosis, showing poor pharmacological tolerance (Montazeri et al., 2017). Furthermore, current chemotherapy is not able to destroy tissue cysts, and the emergence of *T. gondii* strains resistant to current drugs is ongoing (Montazeri et al., 2018). In the absence of an effective human vaccine to treat toxoplasmosis, the development of novel, safe, and more effective drugs is a real need.

β-Carbolines (βCs) are naturally occurring alkaloids present in a broad spectrum of living species (Dai et al., 2018). In particular, βCs are the most important constituents of the plant *Peganum harmala*, used for generations as folk medicine for the treatment of diverse illnesses, including parasitosis (Moloudizargari et al., 2013). Due to their biological activity against a wide range of protozoans, these compounds represent excellent candidates. For example, harmine (**1**) caused necrosis by a nonspecific membrane damage in *Leishmania donovani* promastigotes and exerted antileishmanial activity both *in vitro* and *in vivo* (Lala et al., 2004). In addition, **1** and harmane were active against both promastigote and amastigote forms of *Leishmania infantum*, whereas harmaline exerted a strong antileishmanial activity toward the intracellular amastigote form, preventing the promastigote internalization within macrophages by inhibiting parasite protein kinase C (Di Giorgio et al., 2004). Norharmane, harmane, and **1** were also effective against *Trypanosoma cruzi* epimastigotes, *in vitro*, by inhibiting the parasite’s respiratory chain (Rivas et al., 1999). Additionally, **1** and some chloro- and bromo-tetrahydro-βC derivatives were reported to act as antiplasmodial agents (Shahinas et al., 2012; Bayih et al., 2016). The quite high binding affinity of the latter βCs and the ATP-binding domain of *Plasmodium* heat shock protein 90 (Hsp90) would be a key step in the mechanism of action. These compounds also showed a significant reduction of parasitemia *in vivo*, exerting a synergistic effect when co-administered with other existing antimalarial drugs (Shahinas et al., 2012; Bayih et al., 2016).

**FIGURE 1:**
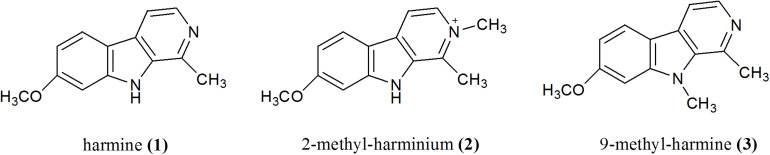
Chemical structures of βCs studied in this work.

In a previous work, we found that **1**, norharmane, and harmane inhibited *T. gondii* invasion and replication in a dose-dependent manner, with **1** being the most effective compound in blocking a parasite’s growth (Alomar et al., 2013). In this context and based on the fact that methyl-substituted βCs show enhanced biological and/or antimicrobial properties (Dai et al., 2018), the *in vitro* antitoxoplasmic activity of 2-methyl-harminium (**2**) and 9-methyl-harmine (**3**) (Figure 1) was evaluated herein. In particular, the effect on parasite invasion, replication, and growth processes was assessed.

## Materials and Methods

### Parasite Source, Culture, and Manipulation

Tachyzoites of the virulent *T. gondii* strains RH Δ*hxgprt* and RH RFP [red fluorescent protein-tagged (Li et al., 2013), kindly provided by Dr. Silvia Moreno, University of Georgia, Athens, Georgia] were propagated in Vero (epithelial kidney *Cercopithecus aethiops*) or hTERT immortalized human foreskin fibroblast cells, incubated with Dulbecco’s modified Eagle medium (DMEM, Gibco BRL) supplemented with fetal bovine serum (FBS, 1% or 10% v/v for Vero and hTERT fibroblasts, respectively), penicillin (100 UI/ml; GIBCO), and streptomycin (100 μg/ml; GIBCO) in a humidified 5% CO_2_ atmosphere at 37°C.

### Chemicals

Compound **1**, SDZ, and CPT were provided by Sigma-Aldrich at the highest purity available (>98%) and were used without further purification. Syntheses of **2** and **3** (purity > 98%) were described previously (Rasse-Suriani et al., 2018). Stock solutions were prepared in DMSO (Biopack) and stored at 4°C. All working solutions were freshly prepared in supplemented DMEM at a final DMSO (vehicle) concentration of 0.5% v/v.

### Cytotoxic Effect of Studied βCs on Host Cells

To evaluate the viability of host cells after treatment with the different compounds, a colorimetric 3-[4,5-dimethylthiazol-2-yl]-2,5-diphenyltetrazolium bromide (MTT) assay (Mosmann, 1983) was performed. Briefly, monolayers of Vero cells, grown in 96-well plates, were exposed to several dilutions of the assayed compounds or the vehicle (DMSO) as negative control, prepared in supplemented DMEM (see above). After 2 days of incubation, the medium was removed, cells were washed once with phosphate-buffered saline (PBS), and treated with 0.5 mg/ml MTT (Sigma-Aldrich, 100 μl per well) in PBS, for 2 h, at 37°C and 5% CO_2_. Supernatants were newly removed, and formazan crystals were solubilized in DMSO (100 μl per well).

The same assay was performed for hTERT fibroblasts, incubated for 4 days with different concentrations of **3**. Absorbance was read at 540 nm (reference wavelength: 700 nm) in a plate reader (Synergy H1 Hybrid Reader, BioTek). Average absorbance from background wells (without cells, treated as the samples) was subtracted out, to obtain corrected values (A540 corr). The percentage of cell viability was calculated as follows:

$$Cellviability\left(\%\right)=\frac{A\,540\,c\,o\,r\,r\,t\,r\,e\,a\,t\,e\,d\,c\,e\,l\,l\,s}{A\,540\,c\,o\,r\,r\,u\,n\,t\,r\,e\,a\,t\,e\,d\,c\,e\,l\,l\,s}\times 100$$

CC50 values were obtained by nonlinear regression analysis of cell viability (%) *vs.* log[compound] with a variable Hill’s slope, using the GraphPad Prism 5.03 software. Each concentration was assayed in triplicates, in three independent sets of experiments. To avoid effects of evaporation, outer wells were not used.

### Invasion Assay

Extracellular tachyzoites of RH Δ*hxgprt* strain (2 × 10^6^) were preincubated for 1 h (at 37°C and 5% CO_2_) with different doses of the assayed compounds (up to 40–50 μM) or the vehicle (DMSO) diluted in supplemented DMEM. Parasites were, then, centrifuged, resuspended in supplemented DMEM, and were allowed to invade Vero cell monolayers grown on coverslips in 24-well plates by incubation for 10 min on ice followed by 1 h at 37°C. Invasion assay was performed as described elsewhere (Yañuk et al., 2017). To facilitate counting, cells were analyzed by indirect immunofluorescence assay (IFA). Immunostaining was performed using anti-SAG1 mouse monoclonal (Novus Biologicals, 1:50)/goat anti-mouse Alexa Fluor 488 (Invitrogen, 1:4,000) antibodies. Fifty random fields per sample, with a similar number of host cells, were analyzed under microscope (Zeiss Axio Observer Colibri 7, magnification × 63). The number of parasitophorous vacuoles per field (PV/field) was counted. Data are presented as means of PV/field ± SEM. Two independent experiments were performed in duplicates.

### Replication Assay

The effect of βCs on the replication process was evaluated on both extracellular and intracellular treated tachyzoites of the strain RH Δ*hxgprt*. In the first case, the procedure followed was described above (see invasion assay), but after allowing the invasion, samples were washed with PBS and incubated for 1 day with supplemented DMEM (without drugs), before fixing and staining. To investigate βCs’ action on intracellular parasites, confluent Vero cells grown in 24-well plates were infected with 2 × 10^6^ tachyzoites per well and treated for 2 days with different concentrations of the investigated drugs or the vehicle (DMSO) as negative control. Finally, they were fixed and immunolabeled as detailed previously. The number of tachyzoites per PV was analyzed under a microscope with × 63 magnification. At least 100 PVs selected at random were scored in triplicates, per compound dose, in two independent sets of experiments. Results are depicted as the mean percentage of PVs (% PV) that contained a geometric progression 2^n^ (with “n” being a natural number ≥ 0) of tachyzoites per PV ± SEM.

### Cell Cycle Analysis

Tachyzoites of the RH Δ*hxgprt* strain (3 × 10^6^) were added to hTERT cell monolayers grown into six-well plates. After invasion was allowed, the medium was changed, and infected cells were incubated for 2 days with **1**, **2**, and **3** (7.5 μM); SDZ (500 μM, positive control); or the vehicle (negative control), diluted in supplemented DMEM. Then, cells were detached using trypsin/EDTA solution and gently ruptured by successive passages through 27- and 30-gauge needles in order to obtain free parasites. Samples were centrifuged (2,000 rpm, 10 min), washed with PBS, fixed with 70% ethanol, and stored for 1 day at −20°C. Afterward, samples were processed and stained as described previously (Munera Lopez et al., 2019). At least 10,000 tachyzoites per sample were analyzed by flow cytometry measurements, carried out in a BD FACSCalibur equipment. Results were analyzed using FlowJo software 7.6. All assays were performed in duplicates, in two independent experiments.

### Lytic Cycle

Tachyzoites (1 × 10^4^) of the RH RFP strain were allowed to invade hTERT fibroblast monolayers grown into 96-well plates, by incubation for 10 min on ice and 1 h at 37°C and 5% CO_2_. Then, cells were washed with PBS and treated with different concentrations of the investigated drugs (up to 10 μM), SDZ (250 and 500 μM) as positive control, or the vehicle (DMSO) as negative control diluted in supplemented DMEM (200 μl per well). Fluorescence measurements were carried out at 3 d.p.i. (and also at 4 d.p.i. for **3**) using a Synergy H1 Hybrid Reader (BioTek). Samples were excited at 544 nm, and fluorescence emission was collected at 590 nm from the bottom (gain: 150). Data were obtained in triplicates, in two independent experiments, and fluorescence values (expressed as relative fluorescence intensity units or RFU) were corrected by subtracting the respective average RFU of background wells (containing samples without parasites but treated in the same manner). IC50 values were obtained by nonlinear regression analysis of % RFU normalized to negative controls vs. log[**3**] with a variable Hill slope, using GraphPad Prism 5.03 software. Outer wells were not used, in order to prevent effects of evaporation.

### Plaque Reduction Assay

Monolayers of hTERT fibroblasts grown into 24-well plates were infected with 1 × 10^4^ tachyzoites per well of the RH Δ*hxgprt* strain. Then, they were washed with PBS and treated with different concentrations of **3** or the vehicle (DMSO) diluted in supplemented DMEM. After 6.5 days of incubation, monolayers were washed, fixed with ethanol (70% v/v), and stained with crystal violet (Sigma). The IC50 value was obtained by nonlinear regression analysis of the number of plaques vs log[**3**] (variable Hill’s slope). The area of the plaques was analyzed by ImageJ software.

### Time of Removal Assay

Monolayers of hTERT cells, grown in 96-well plates, were infected with 1 × 10^4^ tachyzoites of the strain RH RFP. Afterward, cells were washed with PBS and treated with 5, 7.5, and 10 μM of **3** or the vehicle (DMSO), diluted in supplemented DMEM (200 μl per well). Finally, the medium was replaced by fresh DMEM without drugs after 0.7, 1.7, 2, or 15 days of incubation, and fluorescence emission data were collected at different times, up to 15 d.p.i., as was detailed for lytic cycle experiments. RFU values were corrected by subtracting the average RFU of appropriate background wells (with the same treatment to that of samples, but without parasites). Results were obtained in triplicates.

### Western Blot

Monolayers of hTERT cells grown in 12-well plates were infected with 5 × 10^6^ tachyzoites of the RH Δ*hxgprt* strain. After the invasion process, cells were washed with PBS and incubated with **3** (7.5 μM), CPT (10 μM), **3** (7.5 μM) plus CPT (10 μM), or the vehicle (DMSO), diluted in supplemented DMEM for 2 days. Then, infected cells were harvested by trypsinization, centrifuged, resuspended in PBS, and lysed by freeze–thaw cycling. Samples were resolved by SDS-PAGE (15%), and a western blot assay was conducted. Membranes were incubated (1 h, RT) with rabbit anti-*T. gondii* γH2A.X (1:500) (Contreras et al., 2021) and murine anti-SAG1 (Novus Biologicals, 1:500). Finally, they were washed several times with PBS-T and incubated with alkaline phosphatase-conjugated anti-rabbit or anti-mouse secondary antibodies, diluted to 1:4,000 (Santa Cruz Biotechnology). Immunoreactive protein bands were visualized by the NBT-BCIP (Promega) method. Densitometric analysis of scanned images was performed using the Image-Pro Plus software.

### Statistical Analysis

Data were analyzed by one-way ANOVA, followed by Dunnett’s test or Kruskal–Wallis one-way ANOVA on ranks followed by Dunn’s method, depending on the data set characteristics. Results were considered significant for *p* < 0.05. GraphPad Prism 5.03 was used to this end.

## Results

### Cytotoxicity of 1–3 on Vero Cells

To establish the operational concentration range for the antiparasitic studies, cytotoxicity of **1**–**3** was tested on host cells (Vero). Cell viability was evaluated by MTT assay after 2 days of treatment under similar conditions used for *T. gondii* replication studies and cell cycle assays (see below). Data obtained from dose–response curves (Supplementary Figure 1) revealed that neutral compounds **1** and **3** were more cytotoxic than the quaternary derivative, **2**, with CC50 values of 57 (±4) μM, >500 μM, and 59 (±8) μM for **1**, **2**, and **3**, respectively. In addition, under identical experimental conditions, SDZ showed a CC50 value of >500 μM (Table 1).

**TABLE 1:** Cytotoxicity and anti-*T. gondii* activity of compounds **1**–**3**.

	1	2	3	SDZ	Pyrimethamine
CC50 (μM)	57 ± 4^a^ 49 ± 2^b^	>500^a^	59 ± 8^a^ 31 ± 2^b^	>500^a^	>40.2^c^
IC50 (μM)	>10^d,e^	>10^d^	1.6 ± 0.2^d^ 1.8 ± 0.2^e^ 3.1 ± 0.9^f^	∼500^d^ 2,397–2,797^g^ 307.7^h^	3.4^c^ 0.8^g^
SI	<5	–	17.2	–	>11.9^c^

*^*a,b*^Cytotoxic effects of compounds tested on Vero cells and hTERT fibroblasts after 2 and 4 days of incubation, respectively. Values listed are the mean ± SEM of three independent experiments in triplicates.*

*^*c*^Data reported for RH strain and HFF host cells (Abugri et al., 2018).*

*^*d,e,f*^IC50 values obtained by RFP assay after 3 and 4 days of incubation and by plaque assay after 6.5 days of incubation, respectively. Results are means ± SEM of two independent experiments in triplicates.*

*^*g*^Data reported for RH strain (van der Ven et al., 1996).*

*^*h*^Data reported for RH strain (Doliwa et al., 2013).*

### Effects of 1–3 on Extracellular and Intracellular Tachyzoites

**FIGURE 2:**
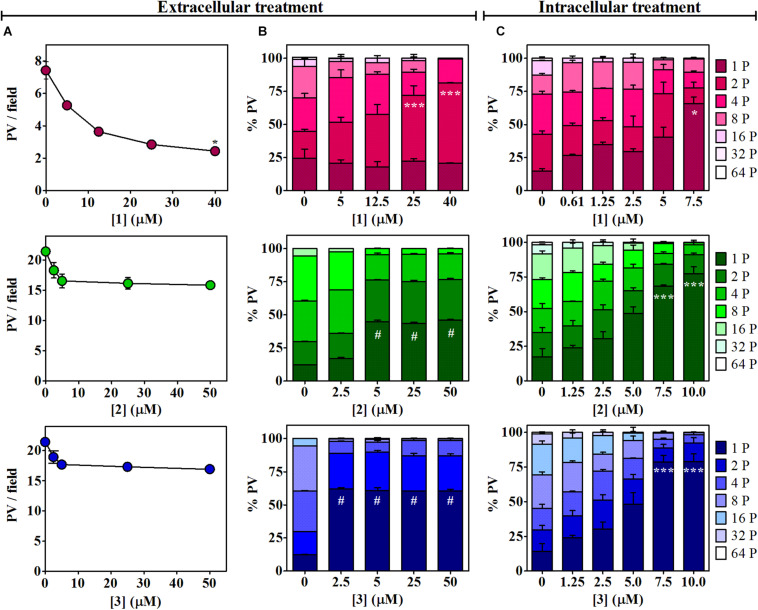
Effects of **1**–**3** on *Toxoplasma* invasion and replication. **(A)** Invasion process. Extracellular tachyzoites, treated with different doses of βCs or the vehicle (DMSO) for 1 h, were allowed to invade Vero cell monolayers. Data are presented as means of PV/field ± SEM. Graphs are representative of two independent experiments performed in duplicates. **(B,C)** Replication process. Percentage of PV with 1, 2, 4, 8, 16, 32, or 64 tachyzoites (from dark-colored to light-colored stacked bars) were obtained for **(B)** 1-h-pretreated parasites after 1 day of incubation and **(C)** intracellular tachyzoites after 2 days of incubation with different concentrations of the investigated βCs or the vehicle. Results are expressed as means of % PV ± SEM obtained from two independent experiments performed in triplicates. Data were analyzed by Kruskal–Wallis/Dunn’s (asterisks) or ANOVA/Dunnett’s (hash symbols) tests. Marks indicate significant differences in PV/field or in % PV having one parasite [or two parasites for **1** in column **(B)**] between treated samples and controls (*^,#^*p* < 0.05; ***p < 0.001). For **1**, results in columns **(A,B)** were previously published by Alomar et al. (2013) and were included here for comparative purposes.

The effect of βCs on the invasion and replication capability of *T. gondii* was then evaluated. To this end, extracellular tachyzoites were preincubated (1 h) with different βC concentrations ranging from 0 up to ∼50 μM. Results depicted in Figure 2 (column A) show that the invasion process was inhibited when parasites were pretreated with **1**. On the contrary, a negligible or rather low effect was exerted by compounds **2** and **3**. Also, the replication process was affected after a pretreatment with the three drugs (Figure 2, column B). Briefly, in the range of 5–50 μM, **2** and **3** produced around fourfold and fivefold increases of the percentage of PV with just one parasite (% PV (1P)), whereas tachyzoites pretreated with **1** produced a sharp increase in the percentage of PV with two parasites (% PV (2P)) with respect to the controls only at the higher doses (25–40 μM). Thus, **1** would have a lower and more retarded action than **2** and **3**. It is noteworthy that in the latter assays, the number of PV/field followed the same trend observed in the invasion experiments (data not shown).

The activity of the three studied compounds was also tested in infected Vero cell culture. Experiments were performed at noncytotoxic doses to host cells, according to the dose–response curves (Supplementary Figure 1). Briefly, upon 2 days of incubation of infected cells with **1**, **2**, and **3**, a detrimental effect on tachyzoite replication was clearly observed, in a dose-dependent manner (Figure 2, column C, and Supplementary Figure 2). This effect was also dependent on the chemical structure of the βC tested. Compound **3** was the most effective since at 7.5 μM, this particular drug led to the formation of 78% of PV (1P), whereas **1** and **2** induced the formation of 65 and 68% PV (1P), respectively.

### Effect of βCs in Tachyzoite Cell Cycle

**FIGURE 3:**
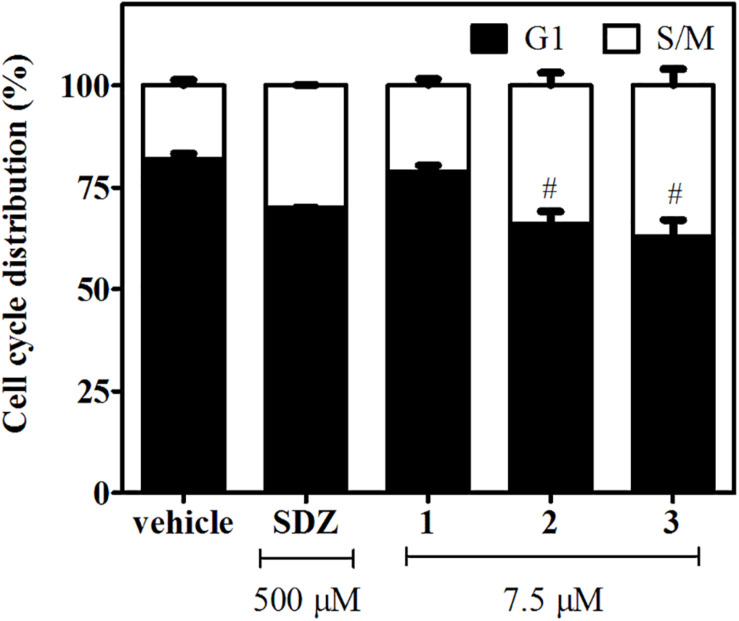
Effects of **1**–**3** (7.5 μM) and SDZ (500 μM) on tachyzoite cell cycle distribution. Results are expressed as the mean percentages of tachyzoites in G1 (black) or S/M (white) phases ± SEM. Hash symbols indicate significant differences between drug treatments and controls, according to ANOVA/Dunnett’s tests (*p* < 0.05).

To this aim, infected hTERT fibroblasts were incubated for 2 days in the presence of **1**, **2**, or **3** (7.5 μM). Treatments with SDZ (500 μM) and the vehicle were used as positive and negative controls, respectively. We found that *N*-methyl-derivatives **2** and **3** produced a significant enrichment in tachyzoite DNA content compatible with S/M phases (Figure 3 and Supplementary Figure 3). A similar behavior was observed for tachyzoites treated with SDZ, which inhibits the dihydropteroate synthase, an important enzyme for pyrimidine biosynthesis in the parasite (Montazeri et al., 2018). On the contrary, a negligible or null effect on intracellular tachyzoites treated with **1** was observed.

### Role and Effect of βCs on the Lytic Cycle of *T. gondii*

**FIGURE 4:**
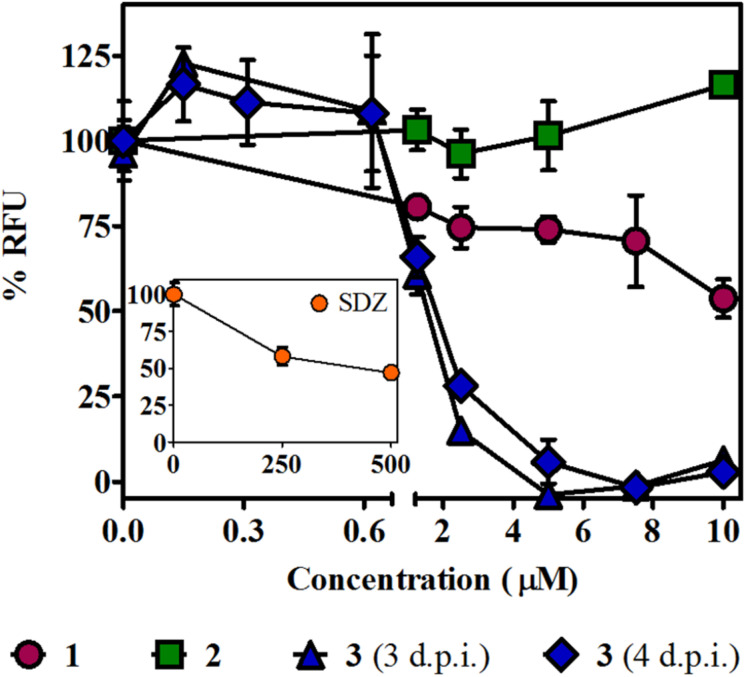
Effect of βCs on the lytic cycle of *T. gondii*. Intracellular tachyzoites (RH RFP strain) were cultured in the presence of studied compounds or the vehicle. Results are means of % RFU (normalized to negative controls) ± SEM obtained after 3 or 4 days of treatment with several concentrations of **1**–**3**. The graph includes results from two independent experiments made in triplicates. Inset: Effect of SDZ (positive control) at the same conditions.

The *T. gondii* lytic cycle is essential for parasite survival within the host cells and, therefore, toxoplasmosis progression (Wang et al., 2020). The effect of the studied βCs on the parasite’s growth was then explored. RFP-expressing tachyzoites were cultured in hTERT fibroblasts, in the presence of the vehicle (negative control), different concentrations of **1–3** or SDZ (positive control), and emission fluorescence measurements were performed at 3 d.p.i. Our results show that compounds **1** and **2** had no major impact on the tachyzoite’s growth (Figure 4), despite **1** producing certain inhibitions of the invasion process and both **1** and **2** inducing significant replication rate reductions. On the contrary, **3** exhibited a strong growth inhibition in a dose-dependent manner, with an IC50 value of 1.6 (±0.2) μM. In particular, for this compound, the same assay was performed at 4 d.p.i., and results revealed that growth inhibition was maintained throughout the experiment period, as depicted in overlaid dose–response curves in Figure 4, and the corresponding IC50 value of 1.8 (±0.2) μM was obtained. Additionally, host cell viability in the presence of different amounts of **3** was evaluated by MTT assay at the later conditions (4 days of incubation). We obtained a CC50 value of 31 (±2) μM and, hence, a calculated selectivity index (SI) of 17.2 (Table 1). It is important to note that at 10 μM (the higher concentration used in the parasite’s growth assays), nonlinear regression analysis of MTT data showed no impact of **3** on fibroblast metabolism (Supplementary Figure 4). Furthermore, although treatment with SDZ 250 and 500 μM produced a decrease in the parasite’s growth (see a decrease of ∼50% in RFU values being reached with respect to untreated controls in the inset of Figure 4), this effect was certainly lower than that achieved after treatment with 5, 7.5, or 10 μM of **3**.

The antiproliferative effect of **3** was also confirmed by plaque reduction assays (Supplementary Figure 5) that yielded an IC50 value of 3.1 (±0.9) μM when tachyzoites of the RH Δ*hxgprt* strain were incubated with different concentrations of **3** for 6.5 days (Table 1). Under these conditions, no parasite growth was observed in cultures treated with 7.5 μM of **3**, whereas untreated wells showed a considerable number of plaques. Interestingly, the analysis of the area of plaques showed the same trend: a decrease in the plaque size as a function of βC concentration.

### Recovery of Parasite Growth After Treatment With 3

**FIGURE 5:**
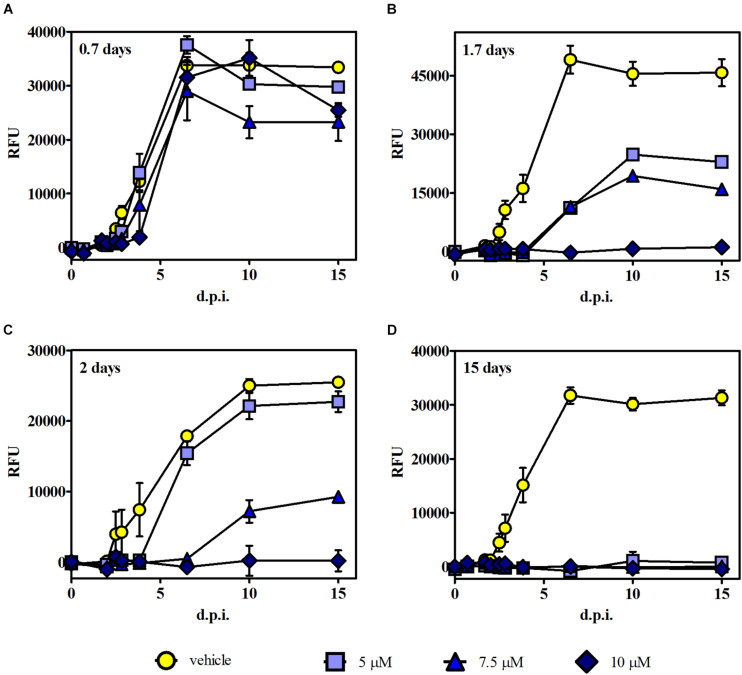
Time of removal assays. Intracellular tachyzoites (RH RFP strain) were cultured in the presence of 5, 7.5, and 10 μM of **3** or the vehicle, and after **(A)** 0.7, **(B)** 1.7, and **(C)** 2 days of treatment, the medium was changed. Parasite growth was monitored by fluorescence emission measurements in the absence of the compound at different times up to 15 d.p.i. **(D)** The compound was present during the whole assay. Results are means (triplicates) of RFU ± SEM.

Time of removal assays were carried out to further analyze whether the growth effect induced by **3** was reversible or irreversible. In these experiments, intracellular RFP-expressing tachyzoites were exposed to **3** at those levels of concentration that reached the higher growth inhibition (i.e., 5, 7.5, and 10 μM) in the growth assay, and after different times post infection, the medium with the drug (or the vehicle in control samples) was replaced by fresh DMEM. Fluorescence measurements were performed up to 15 d.p.i. Results indicated that 0.7 days of treatment was not effective for any compound concentration (Figure 5A). Instead, 1.7 and 2 days of treatment showed a significant reduction of tachyzoite growth for 7.5 μM and total growth inhibition for 10 μM. These data suggest that compound **3** induces an irreversible but concentration- and time-dependent tachyzoite growth inhibition (Figures 5B,C). Additionally, continuous treatment with **3** (up to 15 days) resulted in a complete inhibition of parasite growth (Figure 5D).

It is noteworthy that in this experiment, the plateau reached in RFU values starting around 6 d.p.i. for controls and those ineffective conditions correlates with extended lysis of the host cells. Furthermore, fluorescence intensity results were in agreement with MTT data obtained from the same culture plates at 15 d.p.i.; i.e., the highest fluorescence intensity matched with the lowest absorbance value (Supplementary Figure 6), indicating cell monolayer break due to the progression of lytic cycle.

### Effect of Compound 3 on *Toxoplasma* H2A.X Phosphorylation

**FIGURE 6:**
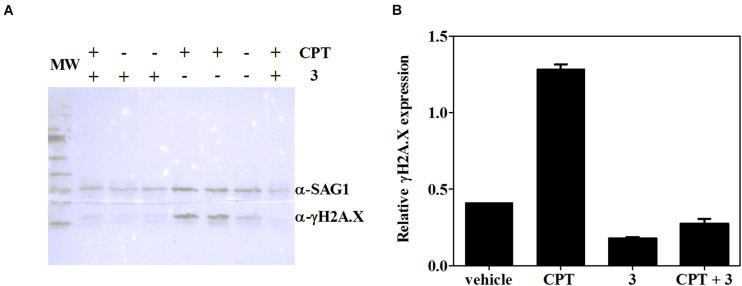
**(A)** Expression of *T. gondii* γH2A.X evaluated by western blot after 2 days of treatment with CPT (10 μM), **3** (7.5 μM), CPT (10 μM) + **3** (7.5 μM) or the vehicle (DMSO). **(B)** Density measurements of western blot bands, relativized to SAG-1 expression.

It has been well documented that βCs are able to inhibit key enzymes including topoisomerases I (Cao et al., 2005) and II (Deveau et al., 2001) and also certain DNA repair enzymes such as phage T4-induced UV endonuclease (Warner et al., 1981). In this context, and considering that the studied compounds are able to arrest *T. gondii*’s cell cycle in S/M phases, the influence of **3** (the most effective compound) on γH2A.X levels was further evaluated herein. The accumulation of this phosphorylated protein reveals double-strand break (DSB) formation, one of the most damaging types of DNA damage (Shrivastav et al., 2008; Fenoy et al., 2016), albeit other replication-associated defects may also trigger γH2A.X activation (Munera Lopez et al., 2019). To this aim, intracellular tachyzoites were incubated with **3** (7.5 μM), CPT [10 μM, which corresponds to two times the reported IC50 value of this compound for RH and RH RFP strains (Munera Lopez et al., 2019)], or a mixture of **3** and CPT (7.5 and 10 μM, respectively) for 2 days. Western blot assay was performed afterward. As depicted in Figure 6, *Toxoplasma* tachyzoites cultured *in vitro* showed detectable basal levels of γH2A.X (Dalmasso et al., 2009; Nardelli et al., 2013; Munera Lopez et al., 2019), due to their high DNA replication rates that lead to replication stress (Munera Lopez et al., 2019). Also, CPT treatment yielded a notable increase of γH2A.X levels, as expected for an inhibitor of topoisomerase I, that produces fork collapse and generates DSB (Rybak et al., 2016). Finally, treatment of intracellular tachyzoites with **3** not only decreased basal levels of γH2A.X but also reduced them significantly when it was co-administered with CPT.

## Discussion

The current pharmacological treatments of toxoplasmosis have strong clinical limitations (Mui et al., 2005; McLeod et al., 2006; Rajapakse et al., 2013; Montazeri et al., 2017, 2018). Hence, the search for novel and safe anti-toxoplasmic drugs is an urgent need. Evaluation of βCs, a family of alkaloids with a broad spectrum of antimicrobial action (Dai et al., 2018), could reap benefits for the ongoing search for effective treatments against the devastating effects of apicomplexan infection. This context led us to study the *in vitro* effect of three harmine derivatives against *T. gondii*.

It is known that *T. gondii* and βCs might exert antagonistic effects on the host cells. On one hand, this parasite stimulates the PI3K/Akt signaling pathway in ARPE-19 cells, to reduce both NADPH oxidase 4 (Nox4) expression and intracellular reactive oxygen species (ROS) levels, generating an optimal microenvironment for its growth (Zhou et al., 2013). On the other hand, βC alkaloids and, in particular, **1** depress the PI3K/Akt/mTOR signaling pathway, which results in autophagy of the insect *Spodoptera frugiperda* Sf9 cell line (Cui et al., 2019). In addition, *N*(2)-benzyl-β-carboline derivative induces cell apoptosis through suppression of PI3K/Akt signaling in HepG2, A549, and HeLa cells (Zhang et al., 2016). With this in mind, extracellular parasites were first treated with each βC (**1**–**3**) prior to cell infection. Data showed that pretreatment with the drugs led to a significant reduction in tachyzoite replication. The latter effect was more evident for **2** and **3**. In contrast, pretreatment with **1** produced a higher decrease in invasion rate. These results confirm that, despite any putative effect that might affect the host cell, βC alkaloids clearly exert a direct effect on the parasite. A similar behavior has been previously described for other related βCs including norharmane and harmane (Alomar et al., 2013).

Since extracellular tachyzoite is not a replicative stage, the pretreatment success could be due to (i) the residual βCs accumulated into the parasites that might act when the replication process occurs and/or (ii) an alteration of the parasite fitness needed to recover after invasion. Interestingly, in a recent work, Denofrio et al. (2020) demonstrated that related βCs accumulate mainly into the mitochondria of HeLa cells after a short incubation time (20 min), and upon longer incubation times, **1** might also accumulate in other intracellular components (Vignoni et al., 2014). It is noteworthy that atovaquone, a drug currently under use for human acute toxoplasmosis treatment, exerts its anti-*T. gondii* action by inhibiting the mitochondrial electron transport processes (McFadden et al., 2000).

Prior to studying the effect of the drugs on intracellular tachyzoites and to dismiss apparent antiparasitic effects induced by the loss of host cell viability, the intrinsic cytotoxicity of these three alkaloids was evaluated against host cells. Our results revealed that neutral compounds **1** and **3** were more cytotoxic (CC50 ∼ 60 μM) than compound **2** (CC50 > 500 μM) when Vero cells were treated with these drugs for 2 days (Table 1). Interestingly, a similar trend has been previously described for the cytotoxicity of other related βCs (i.e., norharmane and harmane) and their respective 9-methyl-derivatives on Vero (Gonzalez et al., 2018) and HEK (Wernicke et al., 2007) cell lines. On the other hand, a marked cytotoxicity reduction observed for the quaternary derivative **2** was also reported for other related quaternary βCs on cells lacking a dopamine transporter (Wernicke et al., 2007).

Finally, our data demonstrated that **1**–**3** were able to decrease the *T. gondii* replication rate, when intracellular tachyzoites (i.e., infected Vero cells) were exposed to alkaloids. However, only **3** was able to strongly abolish the parasite’s growth. The latter effect cannot be ascribed to the difference rate or dynamic of βC release from the cells, since it was recently proven that, after washing, quaternary alkaloids (such as **2**) remain inside the cell longer than the neutral derivatives such as **1** and **3** (Denofrio et al., 2020). It is noteworthy that **3** was also more effective than the gold standard SDZ, used herein as positive control, even at concentration levels 100 times lower. Additionally, the effect shown by compound **3** is comparable to the IC50 value reported for pyrimethamine on the RH strain, ranging from 0.8 to 3.4 μM, depending on the experimental condition (Table 1). Recovery experiments demonstrated that **3** exerted an irreversible action on the tachyzoite’s growth, but in a concentration- and time-dependent manner. These results show that methylation of the harmine skeleton at position N(9) is crucial for anti-toxoplasma effects. Note that other biological effects including cytotoxicity against different cancer cell lines (Cao et al., 2004) and antiviral activities (Gonzalez et al., 2018; Quintana et al., 2020) are also enhanced by 9-methylation of the β-carboline skeleton.

To delve into the mechanism by which compounds **1**–**3** act against *T. gondii* tachyzoites, the progression of parasite cell cycle in the presence of the studied compounds was evaluated, since different βCs have proven to induce cell death through arresting cell cycle at different phases or transitions between them, in different cancer cell lines, yeast (Ahmad et al., 2020), and parasites (Di Giorgio et al., 2004). According to our results, **2** and **3** produced an increase of tachyzoite population in S/M phases, which agree with reported data for related compounds. For instance, **1** induces cell cycle arrest of hepatoma (Zhang et al., 2015) and SW620 cells (Liu et al., 2016) in S and G2/M phases. The same behavior was found for SCG-7901 cancer cells upon treatment with harmaline (Wang et al., 2015) and for *L. infantum* promastigotes treated with **1** and harmane (Di Giorgio et al., 2004). Compound **1** did not show a significant effect, probably due to the low concentration range tested.

High speed in cell division, as occurs in cancer cells (Halazonetis et al., 2008) or tachyzoites, needs high rates of DNA replication, which are associated with replication stress and collapsed replication forks that lead to DNA DSB. This kind of lesion is extremely toxic, and a failure or delay in its repair may result in cell death. Therefore, cells initiate the DNA damage response (DDR) signaling, mediated by three members of the phosphatidylinositol 3′-kinase (PI3K)-like kinase (PIKK) family: DNA-dependent protein kinase (DNA-PK), ataxia telangiectasia mutated kinase (ATM), and ATMRas-3-related kinase (ATR) (Fenoy et al., 2016). Both DNA-PK and ATM are involved mainly in DSB repair, whereas ATR responds to single-stranded DNA structures, associated with resected DNA DSB or stalled replication forks (Mukherjee et al., 2019). These kinases, present in *T. gondii* (Fenoy et al., 2016; Angel et al., 2020), activate by phosphorylating several proteins implicated in DDR, such as histone H2A.X, which is phosphorylated at a SQE motif becoming γH2A.X, an early DBS marker. γH2A.X recruits repair factors near DSB sites and also plays a role in constraining the broken DNA ends (Dickey et al., 2009). Recently, Munera Lopez et al. (2019) demonstrated that ATM mediates H2A.X phosphorylation in response to DSB produced by CPT in *T. gondii* tachyzoites, and such phosphorylation was reduced in the presence of the specific ATM inhibitor KU-55933. Our results also showed that the γH2A.X increase caused by CPT was drastically reduced in a joint treatment with CPT and **3**. Then, the latest compound would be altering the ATM function, as well as other PI3Ks that also phosphorylate the SQE motif of H2A.X (i.e., ATR or DNA-PK) under DSB. Additionally, basal levels of γH2A.X decreased in the presence of **3**. Although other targets can also be affected, the latter results suggest a connection between compound **3** and the alteration of the DNA damage response of *T. gondii*. Moreover, the specific ATM inhibition by KU-55933 leads to tachyzoite cell cycle arrest in the G1 phase (Munera Lopez et al., 2019), as occurs when cancer cells are treated with the same compound (Li and Yang, 2010). Since cell cycle analysis showed a clear increase in S/M phases when **2** and **3** are present, their inhibitory effect may indicate that other DDR effectors could be implicated. Nevertheless, further analysis should be done to find other drug targets. In this regard, cell cycle arrest in G2/M phases occurs, for example, when both ATM and ATR are simultaneously inhibited in HeLa cells treated with chemical DSB inducers (Lima et al., 2016) and also when DNA-PK is specifically inactivated by NU7026 in γ-irradiated ATM-deficient AT5BIVA cells (Shang et al., 2010).

It is well known that βCs bind to DNA molecules. Particularly, previous studies demonstrated that **1**, **2**, and **3** partially intercalate into ctDNA with overall binding constants of 7.7 ± 0.2 (Yañuk et al., 2018), 5.9 ± 0.3 (Denofrio et al., 2020), and 16.8 ± 0.5 (Vignoni et al., 2014) × 10^3^ M^–1^ in bp, respectively, under physiological pH conditions (pH 7.4). However, as most intercalating agents, they do not induce DSB (or other DNA damages) in extracellular DNA (Gonzalez et al., 2012; Denofrio et al., 2020). Although the *in vivo* interaction could affect the topoisomerase activity (Tomczyk and Walczak, 2018), reported data show that **1** and **3** do not inhibit the human topoisomerase II at 600 μM. In addition, albeit topoisomerase I activity is inhibited by **1** and **3** at 150 μM, the latter effect strongly decreases at 50 μM in a cell-free system (Cao et al., 2005). It is important to note that these levels of concentrations are higher than the corresponding doses used herein for the intracellular tachyzoite treatments. Even though these compounds could act at DNA damage and/or DNA repair level, all these evidences suggest that βCs would exert their action impairing DNA repair pathways. In agreement with this argument, Zhang et al. demonstrated that **1** blocks homologous recombination by inhibiting Rad51 recruitment, causing death of hepatoma cells (Zhang et al., 2015). Moreover, **1** and several harmine derivatives do not induce any damage to the DNA integrity in A549 cells (Geng et al., 2018). Nevertheless, further research is needed to assess and to unambiguously elucidate the role of **1**–**3** at the DNA level in *T. gondii* tachyzoites.

In summary, data reported in this work clearly demonstrate that smooth chemical modifications, i.e., 9-methylation, represent a key tool to enhance the antitoxoplasmic activity of this family of alkaloids. Briefly, a promising harmine derivative, showing irreversible action on *T. gondii*’s growth, was identified herein. Data may suggest that compound **3** would affect the DNA repair machinery of the parasite, which is an important source of therapeutic targets. However, further research is still needed to better understand and confirm the mechanism of action.

## Data Availability Statement

The original contributions presented in the study are included in the article/Supplementary Material, further inquiries can be directed to the corresponding author/s.

## Author Contributions

MA, JY, SA, MG, and FC designed the experiments. MA, JY, and MG performed the experiments and analyzed data. MA, SA, MG, and FC drafted the manuscript. All authors read and approved the submitted version.

## Conflict of Interest

The authors declare that the research was conducted in the absence of any commercial or financial relationships that could be construed as a potential conflict of interest.

## Publisher’s Note

All claims expressed in this article are solely those of the authors and do not necessarily represent those of their affiliated organizations, or those of the publisher, the editors and the reviewers. Any product that may be evaluated in this article, or claim that may be made by its manufacturer, is not guaranteed or endorsed by the publisher.

## References

[B1] Abugri D. A. Witola W. H. Russell A. E. Troy R. M. (2018). In vitro activity of the interaction between taxifolin (dihydroquercetin) and pyrimethamine against *Toxoplasma gondii*. *Chem. Biol. Drug Des.* 91 194–201. 10.1111/cbdd.13070 28696589

[B2] Ahmad I. Fakhri S. Khan H. Jeandet P. Aschner M. Yu Z.-L. (2020). Targeting cell cycle by β-carboline alkaloids in vitro: novel therapeutic prospects for the treatment of cancer. *Chem. Biol. Interact.* 330:109229. 10.1016/j.cbi.2020.109229 32835667

[B3] Alday P. H. Doggett J. S. (2017). Drugs in development for toxoplasmosis: advances, challenges, and current status. *Drug Des. Dev. Ther.* 11 273–293. 10.2147/DDDT.S60973 28182168 PMC5279849

[B4] Alomar M. L. Rasse-Suriani F. A. O. Ganuza A. Cóceres V. M. Cabrerizo F. M. Angel S. O. (2013). In vitro evaluation of β-carboline alkaloids as potential anti-Toxoplasma agents. *BMC Res. Notes* 6:193. 10.1186/1756-0500-6-193 23663567 PMC3654986

[B5] Angel S. O. Vanagas L. Ruiz D. M. Cristaldi C. Saldarriaga Cartagena A. M. Sullivan W. J. Jr. (2020). Emerging therapeutic targets against *Toxoplasma gondii*: update on DNA repair response inhibitors and genotoxic drugs. *Front. Cell. Infect. Microbiol.* 10:289. 10.3389/fcimb.2020.00289 32656097 PMC7325978

[B6] Araujo-Silva C. A. De Souza W. Martins-Duarte E. S. Vommaro R. C. (2020). HDAC inhibitors tubastatin A and SAHA affect parasite cell division and are potential anti-*Toxoplasma gondii* chemotherapeutics. *Int. J. Parasitol. Drugs Drug Resist.* 15 25–35. 10.1016/j.ijpddr.2020.12.003 33360687 PMC7771113

[B7] Bayih A. G. Folefoc A. Mohon A. N. Eagon S. Anderson M. Pillai D. R. (2016). In vitro and in vivo anti-malarial activity of novel harmine-analog heat shock protein 90 inhibitors: a possible partner for artemisinin. *Malar J.* 15 579. 10.1186/s12936-016-1625-7 27903279 PMC5131496

[B8] Belk K. Connolly M. P. Schlesinger L. Ben-Harari R. R. (2018). Patient and treatment pathways for toxoplasmosis in the United States: data analysis of the Vizient Health Systems Data from 2011 to 2017. *Pathog. Glob. Health* 112 428–437. 10.1080/20477724.2018.1552644 30526421 PMC6327601

[B9] Cao R. Chen Q. Hou X. Chen H. Guan H. Ma Y. (2004). Synthesis, acute toxicities, and antitumor effects of novel 9-substituted β-carboline derivatives. *Bioorg. Med. Chem.* 12 4613–4623. 10.1016/j.bmc.2004.06.038 15358288

[B10] Cao R. Peng W. Chen H. Ma Y. Liu X. Hou X. (2005). DNA binding properties of 9-substituted harmine derivatives. *Biochem. Biophys. Res. Commun.* 338 1557–1563. 10.1016/j.bbrc.2005.10.121 16288723

[B11] Carruthers V. B. (1999). Armed and dangerous: *Toxoplasma gondii* uses an arsenal of secretory proteins to infect host cells. *Parasitol. Int.* 48 1–10. 10.1016/s1383-5769(98)00042-7 11269320

[B12] Contreras S. M. Ganuza A. Corvi M. M. Angel S. O. (2021). Resveratrol induces H3 and H4K16 deacetylation and H2A. X phosphorylation in *Toxoplasma gondii*. *BMC Res. Notes* 14:19. 10.1186/s13104-020-05416-4 33413578 PMC7792170

[B13] Cui G. Shu B. Veeran S. Yuan H. Yi X. Zhong G. (2019). Natural beta-carboline alkaloids regulate the PI3K/Akt/mTOR pathway and induce autophagy in insect Sf9 cells. *Pestic. Biochem. Physiol.* 154 67–77. 10.1016/j.pestbp.2018.12.005 30765058

[B14] Dai J. Dan W. Schneider U. Wang J. (2018). beta-Carboline alkaloid monomers and dimers: occurrence, structural diversity, and biological activities. *Eur. J. Med. Chem.* 157 622–656. 10.1016/j.ejmech.2018.08.027 30125723

[B15] Dalmasso M. C. Onyango D. O. Naguleswaran A. Sullivan W. J. Jr. Angel S. O. (2009). Toxoplasma H2A variants reveal novel insights into nucleosome composition and functions for this histone family. *J. Mol. Biol.* 392 33–47. 10.1016/j.jmb.2009.07.017 19607843 PMC2734877

[B16] de la Torre A. Pfaff A. W. Grigg M. E. Villard O. Candolfi E. Gomez-Marin J. E. (2014). Ocular cytokinome is linked to clinical characteristics in ocular toxoplasmosis. *Cytokine* 68 23–31. 10.1016/j.cyto.2014.03.005 24787053 PMC4889015

[B17] Denofrio M. P. Rasse-Suriani F. A. O. Paredes J. M. Fassetta F. Crovetto L. Giron M. D. (2020). N-Methyl-beta-carboline alkaloids: structure-dependent photosensitizing properties and localization in subcellular domains. *Org. Biomol. Chem.* 18 6519–6530. 10.1039/d0ob01122c 32628228

[B18] Deveau A. M. Labroli M. A. Dieckhaus C. M. Barthen M. T. Smith K. S. Macdonald T. L. (2001). The synthesis of amino-acid functionalized beta-carbolines as topoisomerase II inhibitors. *Bioorg. Med. Chem. Lett.* 11 1251–1255. 10.1016/s0960-894x(01)00136-6 11392530

[B19] Di Giorgio C. Delmas F. Ollivier E. Elias R. Balansard G. Timon-David P. (2004). In vitro activity of the beta-carboline alkaloids harmane, harmine, and harmaline toward parasites of the species Leishmania infantum. *Exp. Parasitol.* 106 67–74. 10.1016/j.exppara.2004.04.002 15172213

[B20] Dickey J. S. Redon C. E. Nakamura A. J. Baird B. J. Sedelnikova O. A. Bonner W. M. (2009). H2AX: functional roles and potential applications. *Chromosoma* 118 683–692. 10.1007/s00412-009-0234-4 19707781 PMC3094848

[B21] Doliwa C. Escotte-Binet S. Aubert D. Velard F. Schmid A. Geers R. (2013). Induction of sulfadiazine resistance in vitro in *Toxoplasma gondii*. *Exp. Parasitol.* 133 131–136. 10.1016/j.exppara.2012.11.019 23206954

[B22] Dubey J. P. Jones J. L. (2008). *Toxoplasma gondii* infection in humans and animals in the United States. *Int. J. Parasitol.* 38 1257–1278. 10.1016/j.ijpara.2008.03.007 18508057

[B23] Durlach R. A. Kaufer F. Carral L. Hirt J. (2003). Toxoplasmic lymphadenitis-clinical and serologic profile. *Clin. Microbiol. Infect.* 9 625–631. 10.1046/j.1469-0691.2003.00575.x 12925102

[B24] Fenoy I. M. Bogado S. S. Contreras S. M. Gottifredi V. Angel S. O. (2016). The knowns unknowns: exploring the homologous recombination repair pathway in *Toxoplasma gondii*. *Front. Microbiol.* 7:627. 10.3389/fmicb.2016.00627 27199954 PMC4853372

[B25] Flegr J. Prandota J. Sovičková M. Israili Z. H. (2014). Toxoplasmosis-a global threat. Correlation of latent toxoplasmosis with specific disease burden in a set of 88 countries. *PloS One* 9:e90203. 10.1371/journal.pone.0090203 24662942 PMC3963851

[B26] Geng X. Ren Y. Wang F. Tian D. Yao X. Zhang Y. (2018). Harmines inhibit cancer cell growth through coordinated activation of apoptosis and inhibition of autophagy. *Biochem. Biophys. Res. Commun.* 498 99–104. 10.1016/j.bbrc.2018.02.205 29501493 PMC5857254

[B27] Gonzalez M. M. Cabrerizo F. M. Baiker A. Erra-Balsells R. Osterman A. Nitschko H. (2018). β-Carboline derivatives as novel antivirals for herpes simplex virus. *Int. J. Antimicrob. Agents* 52 459–468. 10.1016/j.ijantimicag.2018.06.019 30006037

[B28] Gonzalez M. M. Vignoni M. Pellon-Maison M. Ales-Gandolfo M. A. Gonzalez-Baro M. R. Erra-Balsells R. (2012). Photosensitization of DNA by beta-carbolines: kinetic analysis and photoproduct characterization. *Org. Biomol. Chem.* 10 1807–1819. 10.1039/c2ob06505c 22249177

[B29] Halazonetis T. D. Gorgoulis V. G. Bartek J. (2008). An oncogene-induced DNA damage model for cancer development. *Science* 319 1352–1355. 10.1126/science.1140735 18323444

[B30] Lala S. Pramanick S. Mukhopadhyay S. Bandyopadhyay S. Basu M. K. (2004). Harmine: evaluation of its antileishmanial properties in various vesicular delivery systems. *J. Drug Target.* 12 165–175. 10.1080/10611860410001712696 15203896

[B31] Li Y. Yang D. Q. (2010). The ATM inhibitor KU-55933 suppresses cell proliferation and induces apoptosis by blocking Akt in cancer cells with overactivated Akt. *Mol. Cancer Ther.* 9 113–125. 10.1158/1535-7163.Mct-08-1189 20053781

[B32] Li Z. H. Ramakrishnan S. Striepen B. Moreno S. N. (2013). *Toxoplasma gondii* relies on both host and parasite isoprenoids and can be rendered sensitive to atorvastatin. *PLoS Pathog.* 9:e1003665. 10.1371/journal.ppat.1003665 24146616 PMC3798403

[B33] Lima M. Bouzid H. Soares D. G. Selle F. Morel C. Galmarini C. M. (2016). Dual inhibition of ATR and ATM potentiates the activity of trabectedin and lurbinectedin by perturbing the DNA damage response and homologous recombination repair. *Oncotarget* 7 25885–25901. 10.18632/oncotarget.8292 27029031 PMC5041952

[B34] Liu J. Li Q. Liu Z. Lin L. Zhang X. Cao M. (2016). Harmine induces cell cycle arrest and mitochondrial pathway-mediated cellular apoptosis in SW620 cells via inhibition of the Akt and ERK signaling pathways. *Oncol. Rep.* 35 3363–3370. 10.3892/or.2016.4695 27004568

[B35] Martins-Duarte ÉS. Urbina J. A. de Souza W. Vommaro R. C. (2006). Antiproliferative activities of two novel quinuclidine inhibitors against *Toxoplasma gondii* tachyzoites in vitro. *J. Antimicrob. Chemother.* 58 59–65. 10.1093/jac/dkl180 16702175

[B36] Martynowicz J. Doggett J. S. Sullivan W. J. Jr. (2020). Efficacy of guanabenz combination therapy against chronic toxoplasmosis across multiple mouse strains. *Antimicrob. Agents Chemother.* 64:e00539Ű20. 10.1128/AAC.00539-20 32540979 PMC7449173

[B37] McFadden D. C. Tomavo S. Berry E. A. Boothroyd J. C. (2000). Characterization of cytochrome b from *Toxoplasma gondii* and Q(o) domain mutations as a mechanism of atovaquone-resistance. *Mol. Biochem. Parasitol.* 108 1–12. 10.1016/s0166-6851(00)00184-5 10802314

[B38] McLeod R. Boyer K. Karrison T. Kasza K. Swisher C. Roizen N. (2006). Outcome of treatment for congenital toxoplasmosis, 1981–2004: the national collaborative Chicago-based, congenital toxoplasmosis study. *Clin. Infect. Dis.* 42 1383–1394. 10.1086/501360 16619149

[B39] Moloudizargari M. Mikaili P. Aghajanshakeri S. Asghari M. H. Shayegh J. (2013). Pharmacological and therapeutic effects of *Peganum harmala* and its main alkaloids. *Pharmacogn. Rev.* 7 199–212. 10.4103/0973-7847.120524 24347928 PMC3841998

[B40] Montazeri M. Mehrzadi S. Sharif M. Sarvi S. Tanzifi A. Aghayan S. A. (2018). Drug resistance in *Toxoplasma gondii*. *Front. Microbiol.* 9:2587. 10.3389/fmicb.2018.02587 30420849 PMC6215853

[B41] Montazeri M. Sharif M. Sarvi S. Mehrzadi S. Ahmadpour E. Daryani A. (2017). A systematic review of in vitro and in vivo activities of anti-toxoplasma drugs and compounds (2006-2016). *Front. Microbiol.* 8:25. 10.3389/fmicb.2017.00025 28163699 PMC5247447

[B42] Mosmann T. (1983). Rapid colorimetric assay for cellular growth and survival: application to proliferation and cytotoxicity assays. *J. Immunol. Methods* 65 55–63. 10.1016/0022-1759(83)90303-4 6606682

[B43] Mui E. J. Jacobus D. Milhous W. K. Schiehser G. Hsu H. Roberts C. W. (2005). Triazine inhibits *Toxoplasma gondii* tachyzoites in vitro and in vivo. *Antimicrob. Agents Chemother.* 49 3463–3467. 10.1128/AAC.49.8.3463-3467.2005 16048961 PMC1196210

[B44] Mukherjee S. Abdisalaam S. Bhattacharya S. Srinivasan K. Sinha D. Asaithamby A. (2019). Mechanistic link between DNA damage sensing, repairing and signaling factors and immune signaling. *Adv. Protein Chem. Struct. Biol.* 115 297–324. 10.1016/bs.apcsb.2018.11.004 30798935 PMC7043287

[B45] Munera Lopez J. Ganuza A. Bogado S. S. Munoz D. Ruiz D. M. Sullivan W. J. Jr. (2019). Evaluation of ATM kinase inhibitor KU-55933 as potential anti-*Toxoplasma gondii* agent. *Front. Cell. Infect. Microbiol.* 9:26. 10.3389/fcimb.2019.00026 30815397 PMC6381018

[B46] Nardelli S. C. Che F.-Y. Silmon de Monerri N. C. Xiao H. Nieves E. Madrid-Aliste C. (2013). The histone code of *Toxoplasma gondii* comprises conserved and unique posttranslational modifications. *mBio* 4:e00922-13. 10.1128/mBio.00922-13 24327343 PMC3870261

[B47] Quintana V. M. Selisko B. Brunetti J. E. Eydoux C. Guillemot J. C. Canard B. (2020). Antiviral activity of the natural alkaloid anisomycin against dengue and Zika viruses. *Antivir. Res.* 176:104749. 10.1016/j.antiviral.2020.104749 32081740 PMC7279513

[B48] Rajapakse S. Chrishan Shivanthan M. Samaranayake N. Rodrigo C. Deepika Fernando S. (2013). Antibiotics for human toxoplasmosis: a systematic review of randomized trials. *Pathog. Glob. Health* 107 162–169. 10.1179/2047773213Y.0000000094 23816507 PMC4001466

[B49] Rasse-Suriani F. A. O. García-Einschlag F. S. Rafti M. Schmidt De León T. David Gara P. M. Erra-Balsells R. (2018). Photophysical and photochemical properties of naturally occurring normelinonine F and melinonine F alkaloids and structurally related N(2)- and/or N(9)-methyl-β-carboline derivatives. *Photochem. Photobiol.* 94 36–51. 10.1111/php.12811 28741707

[B50] Rivas P. Cassels B. K. Morello A. Repetto Y. (1999). Effects of some beta-carboline alkaloids on intact *Trypanosoma cruzi* epimastigotes. *Comp. Biochem. Physiol. C Pharmacol. Toxicol. Endocrinol.* 122 27–31. 10.1016/s0742-8413(98)10069-5 10190025

[B51] Rybak P. Hoang A. Bujnowicz L. Bernas T. Berniak K. Zarebski M. (2016). Low level phosphorylation of histone H2AX on serine 139 (gammaH2AX) is not associated with DNA double-strand breaks. *Oncotarget* 7 49574–49587. 10.18632/oncotarget.10411 27391338 PMC5226530

[B52] Shahinas D. Macmullin G. Benedict C. Crandall I. Pillai D. R. (2012). Harmine is a potent antimalarial targeting Hsp90 and synergizes with chloroquine and artemisinin. *Antimicrob. Agents Chemother.* 56 4207–4213. 10.1128/AAC.00328-12 22615284 PMC3421604

[B53] Shang Z. F. Huang B. Xu Q. Z. Zhang S. M. Fan R. Liu X. D. (2010). Inactivation of DNA-dependent protein kinase leads to spindle disruption and mitotic catastrophe with attenuated checkpoint protein 2 Phosphorylation in response to DNA damage. *Cancer Res.* 70 3657–3666. 10.1158/0008-5472.Can-09-3362 20406977

[B54] Shrivastav M. De Haro L. P. Nickoloff J. A. (2008). Regulation of DNA double-strand break repair pathway choice. *Cell Res.* 18 134–147. 10.1038/cr.2007.111 18157161

[B55] Tomczyk M. D. Walczak K. Z. (2018). l,8-Naphthalimide based DNA intercalators and anticancer agents. A systematic review from 2007 to 2017. *Eur. J. Med. Chem.* 159 393–422. 10.1016/j.ejmech.2018.09.055 30312931

[B56] van der Ven A. J. Schoondermark-van de Ven E. M. Camps W. Melchers W. J. Koopmans P. P. van der Meer J. W. (1996). Anti-toxoplasma effect of pyrimethamine, trimethoprim and sulphonamides alone and in combination: implications for therapy. *J. Antimicrob. Chemother.* 38 75–80. 10.1093/jac/38.1.75 8858459

[B57] Vignoni M. Erra-Balsells R. Epe B. Cabrerizo F. M. (2014). Intra- and extra-cellular DNA damage by harmine and 9-methyl-harmine. *J. Photochem. Photobiol. B Biol.* 132 66–71. 10.1016/j.jphotobiol.2014.01.020 24602814

[B58] Wang J. L. Bai M. J. Elsheikha H. M. Liang Q. L. Li T. T. Cao X. Z. (2020). Novel roles of dense granule protein 12 (GRA12) in *Toxoplasma gondii* infection. *FASEB J.* 34 3165–3178. 10.1096/fj.201901416RR 31908049

[B59] Wang Y. Wang C. Jiang C. Zeng H. He X. (2015). Novel mechanism of harmaline on inducing G2/M cell cycle arrest and apoptosis by up-regulating Fas/FasL in SGC-7901 cells. *Sci. Rep.* 5:18613. 10.1038/srep18613 26678950 PMC4683523

[B60] Warner H. R. Persson M. L. Bensen R. J. Mosbaugh D. W. Linn S. (1981). Selective inhibition by harmane of the apurinic apyrimidinic endonuclease activity of phage T4-induced UV endonuclease. *Nucleic Acids Res.* 9 6083–6092. 10.1093/nar/9.22.6083 6273822 PMC327585

[B61] Wernicke C. Schott Y. Enzensperger C. Schulze G. Lehmann J. Rommelspacher H. (2007). Cytotoxicity of β-carbolines in dopamine transporter expressing cells: structure–activity relationships. *Biochem. Pharmacol.* 74 1065–1077. 10.1016/j.bcp.2007.06.046 17692827

[B62] Yañuk J. G. Alomar M. L. Gonzalez M. M. Alonso A. M. Angel S. O. Coceres V. M. (2017). A comprehensive analysis of direct and photosensitized attenuation of *Toxoplasma gondii* tachyzoites. *J. Photochem. Photobiol. B* 177 8–17. 10.1016/j.jphotobiol.2017.10.008 29031212

[B63] Yañuk J. G. Denofrio M. P. Rasse-Suriani F. A. O. Villarruel F. D. Fassetta F. García Einschlag F. S. (2018). DNA damage photo-induced by chloroharmine isomers: hydrolysis versus oxidation of nucleobases. *Org. Biomol. Chem.* 16 2170–2184. 10.1039/c8ob00162f 29528081

[B64] Zhang L. Zhang F. Zhang W. Chen L. Gao N. Men Y. (2015). Harmine suppresses homologous recombination repair and inhibits proliferation of hepatoma cells. *Cancer Biol. Ther.* 16 1585–1592. 10.1080/15384047.2015.1078021 26382920 PMC4846143

[B65] Zhang X.-F. Sun R.-Q. Jia Y.-F. Chen Q. Tu R.-F. Li K.-K. (2016). Synthesis and mechanisms of action of novel harmine derivatives as potential antitumor agents. *Sci. Rep.* 6:33204. 10.1038/srep33204 27625151 PMC5021947

[B66] Zhou W. Quan J.-H. Lee Y.-H. Shin D.-W. Cha G.-H. (2013). *Toxoplasma gondii* proliferation require down-regulation of host Nox4 expression via activation of PI3 Kinase/Akt signaling pathway. *PLoS One* 8:e66306. 10.1371/journal.pone.0066306 23824914 PMC3688893

