# Supplementary material for: In vitro Effect of Harmine Alkaloid and Its N-Methyl Derivatives Against Toxoplasma gondii
Source: Front Microbiol. 2021 Aug 5;12:716534. doi: 10.3389/fmicb.2021.716534 (PMC8375385; doi:10.3389/fmicb.2021.716534)
Supplement: Supplementary file 1 [file Image_1.PDF]

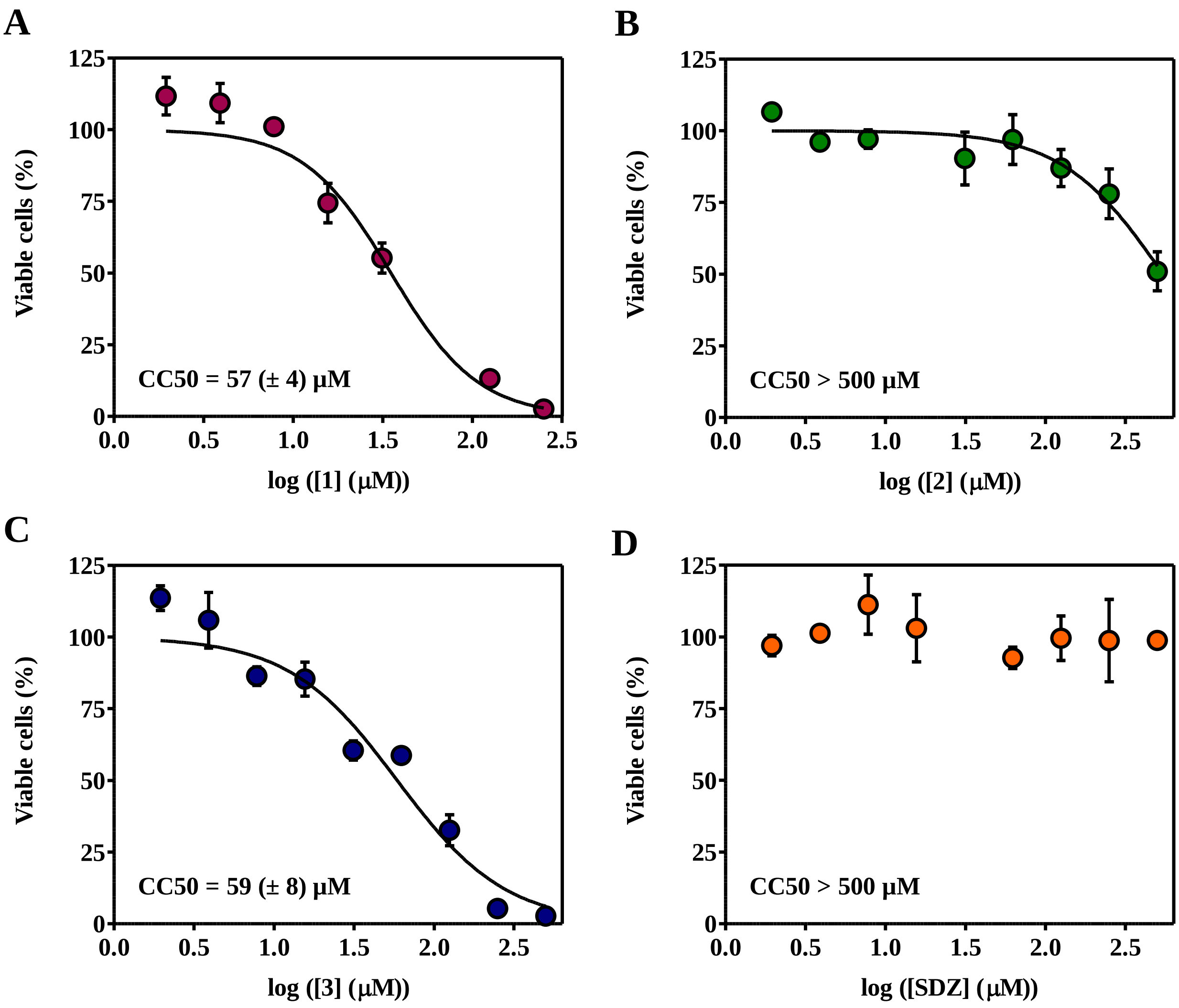

Supplementary Figure 1. Citotoxicity of compounds 1 - 3 and SDZ on Vero cells. Metabolic activity of Vero cells was determined by MTT assay after 2 days of treatment with several concentrations of **(A) 1**, **(B) 2**, **(C) 3** or **(D)** SDZ. CC50 values were obtained by non-linear regression analysis of data from three independent experiments (performed in triplicates).
