# Supplementary material for: In vitro Effect of Harmine Alkaloid and Its N-Methyl Derivatives Against Toxoplasma gondii
Source: Front Microbiol. 2021 Aug 5;12:716534. doi: 10.3389/fmicb.2021.716534 (PMC8375385; doi:10.3389/fmicb.2021.716534)
Supplement: Supplementary file 2 [file Image_2.PDF]

A

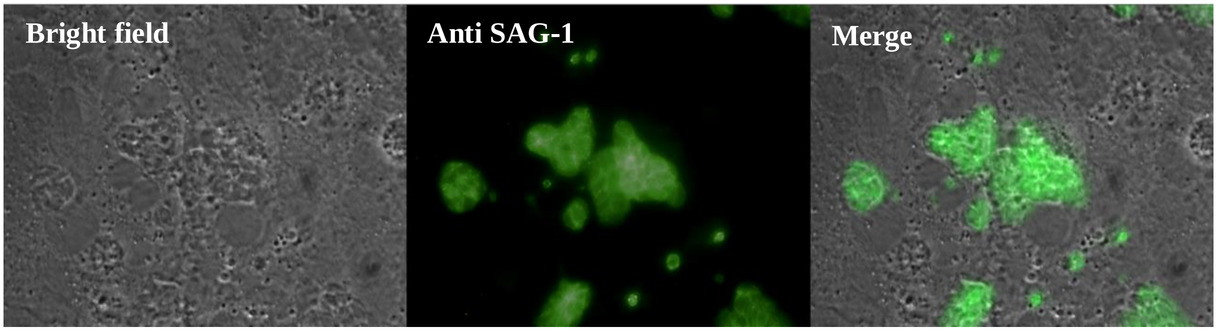

B

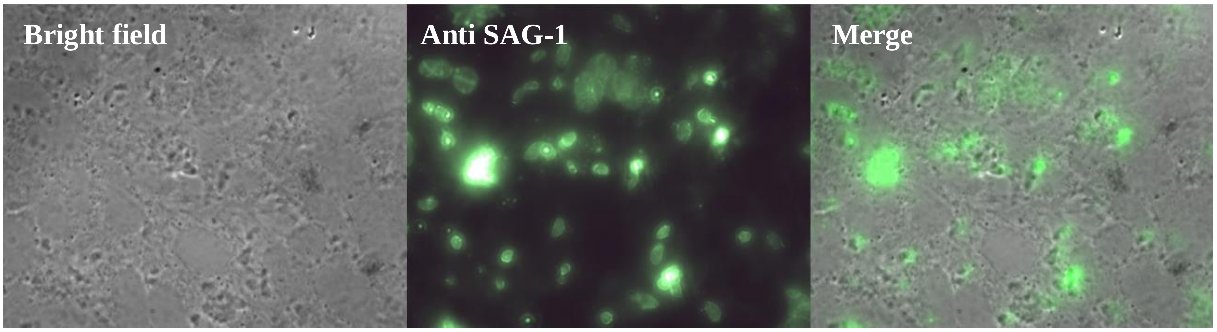

Supplementary Figure 2. Effect of compound 3 on intracellular tachyzoites. Indirect immunofluorescence microscopy images of intracellular tachyzoites treated with **(A)** the vehicle (DMSO) and **(B)** **3** (7.5 µM) during 2 days. Photographs are representative of two independent experiments performed in triplicates.
