# Supplementary material for: In vitro Effect of Harmine Alkaloid and Its N-Methyl Derivatives Against Toxoplasma gondii
Source: Front Microbiol. 2021 Aug 5;12:716534. doi: 10.3389/fmicb.2021.716534 (PMC8375385; doi:10.3389/fmicb.2021.716534)
Supplement: Supplementary file 3 [file Image_3.pdf]

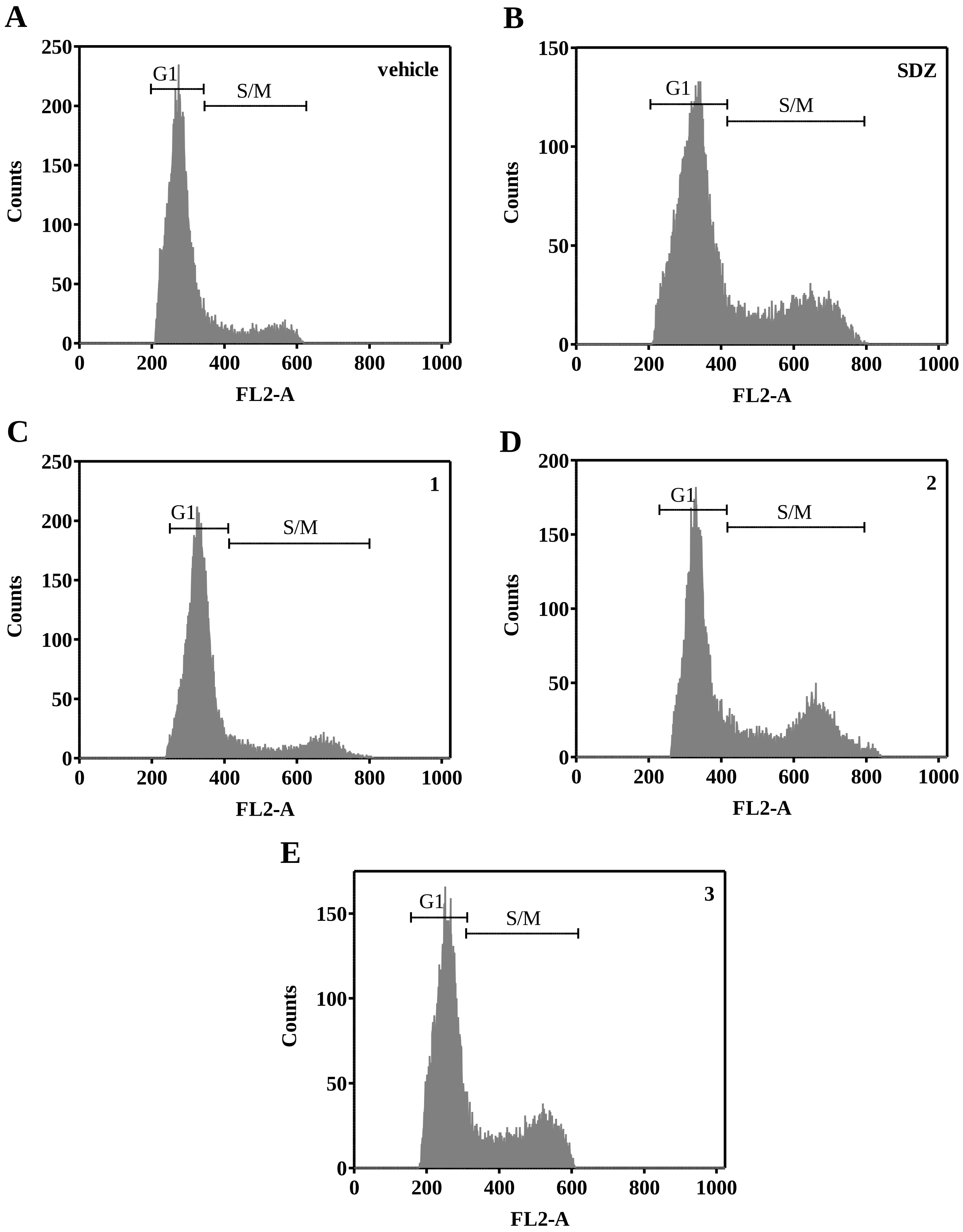

Supplementary Figure 3. Effect of studied compounds in tachyzoite cell cycle. Representative histograms of tachyzoite cell cycle obtained after 2 days of incubation with **(A)** the vehicle (DMSO), **(B)** SDZ 500 µM, **(C) 1**, **(D) 2** and **(E) 3** (all of them 7.5 µM).
