# Supplementary material for: In vitro Effect of Harmine Alkaloid and Its N-Methyl Derivatives Against Toxoplasma gondii
Source: Front Microbiol. 2021 Aug 5;12:716534. doi: 10.3389/fmicb.2021.716534 (PMC8375385; doi:10.3389/fmicb.2021.716534)
Supplement: Supplementary file 4 [file Image_4.PDF]

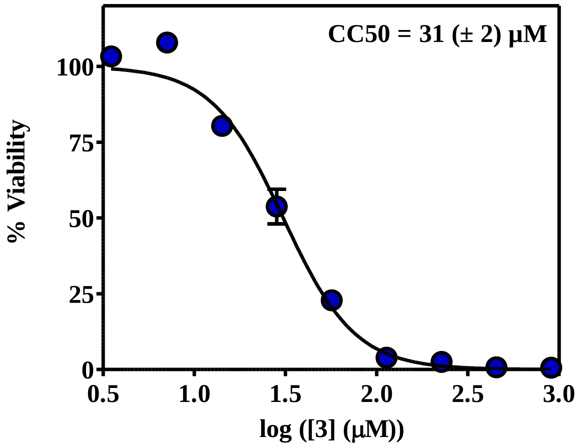

Supplementary Figure 4. Citotoxicity of compound 3 on hTERT fibroblasts. Metabolic activity of hTERT fibroblasts was determined by MTT assay after 4 days of treatment with 2-fold serial dilutions of **3**. A CC50 value of 31 (± 2) µM was obtained by non-linear regression analysis of data from three independent experiments (performed in triplicates).
