# Supplementary material for: In vitro Effect of Harmine Alkaloid and Its N-Methyl Derivatives Against Toxoplasma gondii
Source: Front Microbiol. 2021 Aug 5;12:716534. doi: 10.3389/fmicb.2021.716534 (PMC8375385; doi:10.3389/fmicb.2021.716534)
Supplement: Supplementary file 5 [file Image_5.PDF]

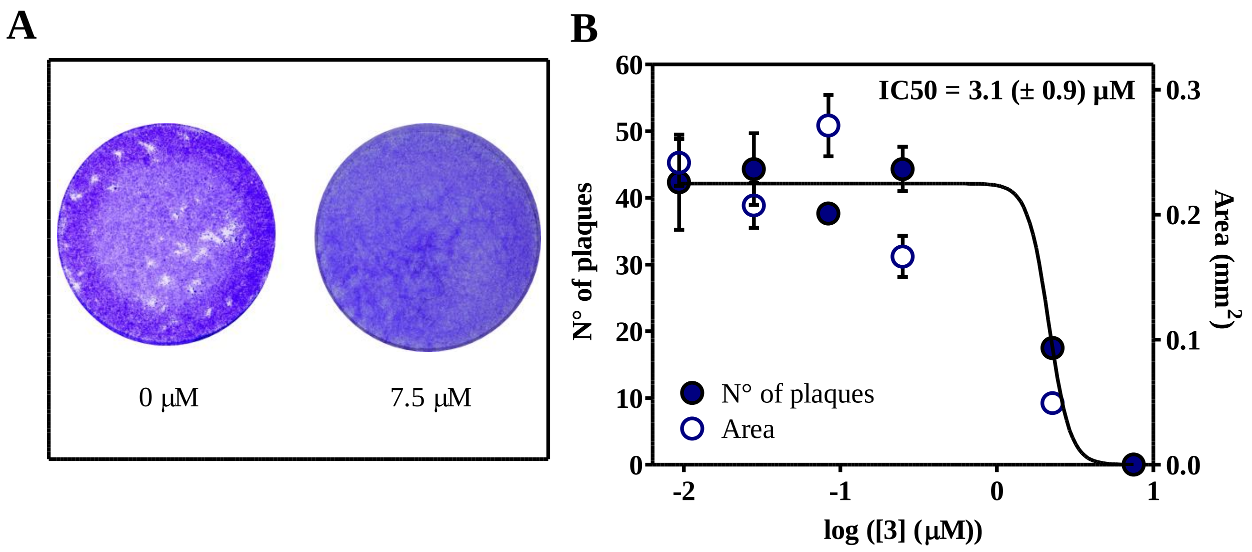

Supplementary Figure 5. Effect of compound 3 in lytic cycle of RH *Δhxgprt* strain. (A) Plaque images of tachyzoites exposed to the vehicle or 7.5 μM of **3** during 6.5 days. **(B)** Number (full circles) and area (open circles) of plaques in infected hTERT fibroblasts as a function of compound **3** concentration. IC50 value was obtained by non-linear regression analysis of number of plaques vs log [**3**] (variable Hill's slope). Both graphs are representative of two independent experiments made in triplicates, with similar results.
