# Supplementary material for: In vitro Effect of Harmine Alkaloid and Its N-Methyl Derivatives Against Toxoplasma gondii
Source: Front Microbiol. 2021 Aug 5;12:716534. doi: 10.3389/fmicb.2021.716534 (PMC8375385; doi:10.3389/fmicb.2021.716534)
Supplement: Supplementary file 6 [file Image_6.PDF]

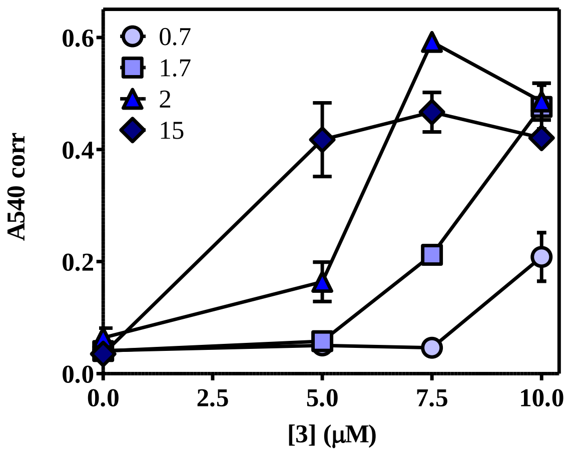

Supplementary Figure 6. MTT assay of infected culture plates. Metabolic activity of infected cell monolayers treated during 0.7, 1.7, 2 or 15 days with **3** at 5, 7.5 or 10 µM, or the vehicle as negative control, measured 15 days post-infection by MTT assay. Data are presented as means of corrected absorbance at 540 nm (A540 corr) ± SEM (reference wavelength = 700 nm).
